# Treatment of B cells malignancies with anti-CD19 CAR+, TCR-, CD52- allogeneic T cells

**DOI:** 10.1186/2051-1426-1-S1-P34

**Published:** 2013-11-07

**Authors:** Cécile Schiffer Mannioui, Laëtitia Lemaire, Laurent Poirot, Agnes Gouble, Sylvain Arnould, Roman Galetto, Julianne Smith, Andrew Scharenberg

**Affiliations:** 1 Cellectis Therapeutics, Paris, France

## 

Encouraging data have emerged from adoptive T-cell therapies in advanced forms of cancer. Anti-tumor immunity is found in tumor infiltrating lymphocytes as well as engineered T cells where exogenous expression of a chimeric antigen receptor (CAR) confers cancer recognition on the cells. Present adoptive immunotherapy methods are restricted to the use of autologous patient T-cells due to the limited persistence of allogeneic T cells and the potential for graft versus host disease (GvHD). The use of autologous patient T cells in cancer immunotherapy is however limited due to the fact that this approach is complex and time consuming. We propose a novel approach to treat B cell malignancies based on the use of genetically modified allogeneic T cells in conjunction with the conditioning regimen alemtuzumab. Allogeneic T cells were engineered to express an anti-CD19 CAR and to no longer express TCRalpha and CD52, responsible for GVHD and the sensitivity to alemtuzumab, respectively. The inactivation of the TCRalpha and CD52 genes in allogeneic T cells was realized by using TALEN^TM^, a novel class of sequence-specific nucleases created by the fusion of transcription activator-like effectors (TALEs) to the catalytic domain of an endonuclease. We have shown that anti-CD19 CAR+ TCR- CD52- allogeneic T cells did not respond to TCR stimulation, were resistant to alemtuzumab treatment and were able to kill target cells expressing CD19 in vitro and in vivo.

